# Hyperactive Cdc2 kinase interferes with the response to broken replication forks by trapping *S.pombe* Crb2 in its mitotic T215 phosphorylated state

**DOI:** 10.1093/nar/gku452

**Published:** 2014-05-26

**Authors:** Salah Adam Mahyous Saeyd, Katarzyna Ewert-Krzemieniewska, Boyin Liu, Thomas Caspari

**Affiliations:** Genome Biology Group, College of Natural Sciences, School of Biological Sciences, Bangor University, Brambell Building, Deiniol Road, Bangor LL57 2UW, Wales, United Kingdom

## Abstract

Although it is well established that Cdc2 kinase phosphorylates the DNA damage checkpoint protein Crb2^53BP1^ in mitosis, the full impact of this modification is still unclear. The Tudor-BRCT domain protein Crb2 binds to modified histones at DNA lesions to mediate the activation of Chk1 by Rad3^ATR^ kinase. We demonstrate here that fission yeast cells harbouring a hyperactive Cdc2^CDK1^ mutation (*cdc2.1w*) are specifically sensitive to the topoisomerase 1 inhibitor camptothecin (CPT) which breaks DNA replication forks. Unlike wild-type cells, which delay only briefly in CPT medium by activating Chk1 kinase, *cdc2.1w* cells bypass Chk1 to enter an extended cell-cycle arrest which depends on Cds1 kinase. Intriguingly, the ability to bypass Chk1 requires the mitotic Cdc2 phosphorylation site Crb2-T215. This implies that the presence of the mitotic phosphorylation at Crb2-T215 channels Rad3 activity towards Cds1 instead of Chk1 when forks break in S phase. We also provide evidence that hyperactive Cdc2.1w locks cells in a G1-like DNA repair mode which favours non-homologous end joining over interchromosomal recombination. Taken together, our data support a model such that elevated Cdc2 activity delays the transition of Crb2 from its G1 to its G2 mode by blocking Srs2 DNA helicase and Casein Kinase 1 (Hhp1).

## INTRODUCTION

Despite the importance of cyclin-dependent kinases (CDKs) for the regulation of the DNA damage response (DDR) (1,2,3), it is still enigmatic how CDKs act as activators of DNA repair while being down-regulated by the DNA damage checkpoint. Two possible answers to this puzzle may lie in the temporal or spatial organization of CDKs.

The activity of CDK1-cyclin B kinase peaks early in mitosis (4) and this rise could prime DDR proteins for their roles in the next cell cycle. Between the end of G2 and the start of the subsequent S phase, cells repair broken chromosomes by non-homologous end-joining (NHEJ) (5,6). To facilitate NHEJ, the Ku70-Ku80 DNA binding complex and the chromatin protein 53BP1^Crb2,Rad9^ prevent the resection of broken DNA ends (7,8,9). It is widely assumed that the transition from NHEJ to homologous recombination (HR) in S phase requires the activity of CDKs. Interestingly, neither progression through S phase nor the presence of the sister chromatid are necessary to initiate HR (10).

Alternative to this temporal regulation, the DNA repair choice could be controlled by CDKs directly at the chromatin. In fission yeast, Cdc2-cyclin B kinase associates with origins of replication on chromosomes during the G1/S transition to prevent reinitiation of S phase (11). Whether this chromosomal pool of Cdc2 also activates HR is currently unknown. In human cells, the Mre11 subunit of the MRN (Mre11-Rad50-Nbs1) complex recruits CDK2-cyclin A to broken chromosomes to promote the formation of a complex between BRCA1 and the endonuclease CtIP^Cip1,Sae2^ (12,13). This BRCA1-CtIP complex stimulates end resection and HR. Such a spatial separation would allow the DNA damage checkpoint to stop progression into mitosis by targeting CDK at the centrosome (14) while leaving the chromosomal CDK pool active to promote DNA repair.

A DDR protein which exhibits an oscillating change in its activity throughout the cell cycle is the BRCT and tudor domain protein 53BP1^Crb2,Rad9^. In G1, human 53BP1 recruits Rif1 to broken DNA to block end resection, a step which is antagonized in S/G2 by BRCA1 in association with CtIP^Ctp1,Sae2^ (13,9,15). After the G1/S transition, 53BP1 turns into an activator to promote the association between the checkpoint kinases ATM^Tel1^ and Chk2^Cds1^ (16). This activator role is later switched off by Polo-like kinase when cells enter mitosis (17). Despite this cyclic regulation, the relationship between human 53BP1 and CDKs is only poorly understood (17). At the mechanistic level, much more is known about the CDK1-dependent regulation of the 53BP1 paralog, Crb2, in fission yeast (*Schizosaccharomyces pombe*). Cdc2 phosphorylates the N-terminus of Crb2 at threonine-215 in mitosis which triggers the formation of a complex between Crb2 and the BRCT domain protein Rad4^Cut5,Dpb11,TopBP1^ on the chromatin (18,19). The cellular activities of the mitotic Crb2-Rad4 complex are not known and are not related to the recruitment of Crb2 to a broken chromosome. The latter process depends on the association of Crb2 with phosphorylated histone H2A through its C-terminal BRCT domains and with K-20 methylated histone H4 through its Tudor domains (20). The mitotic T215 phosphorylation primes, however, the Crb2-Rad4 complex for its rearrangement in G2. Once Cdc2 activity has increased again at the start of G2, Rad4 recruits the kinase to the complex where it modifies a non-canonical Cdc2 site at T187. This enables Crb2 to bind to Chk1, and Rad4 to associate with the Rad9-Rad1-Hus1 checkpoint clamp (21).

Since most studies concerning the DNA repair roles of CDKs were conducted with inhibitors or proteins lacking a CDK phosphorylation site (22), we took advantage of the hyperactive *cdc2.1w* allele in *S. pombe* to investigate how the untimely activation of CDK1^Cdc2^ might affect the DDR. Like most *wee* mutations (‘wee’ cells are short), the *cdc2.1w* (*wee2–1*) mutant enters mitosis prematurely (23,24). The Cdc2.1w kinase carries a glycine-to-aspartate (G146D) replacement in the vicinity of its ATP binding site which renders it insensitive to the inhibition by Wee1 kinase (25). Although *cdc2.1w* and *wee1* deletion cells share the same cell cycle phenotype, only *Δwee1* cells are known to be sensitive to the DNA replication inhibitor hydroxyurea, UV light and ionizing radiation (26,27).

We report here that elevated Cdc2 activity renders *S. pombe* cells specifically sensitive to the topoisomerase 1 poison camptothecin (CPT), an anti-cancer drug which breaks DNA replication forks. While wild-type cells delay mitosis only briefly in G2 when replication forks collapse by activating the Rad3^ATR^-Crb2^53BP1^-Chk1 checkpoint pathway, *cdc2.1w* cells enter a prolonged G2 arrest independently of Chk1. Our data suggest that hyperactive Cdc2.1w traps Crb2 in its G1 mode by blocking Srs2 DNA helicase and Casein Kinase 1 (Hhp1). As this correlates with an increase in NHEJ and a decrease in interchromosomal recombination, the repair of broken replication forks may be delayed leading to the aberrant activation of Cds1 and an extended G2 arrest.

## MATERIALS AND METHODS

### Strains

The following *S.pombe* strains were used in this study: wild-type 804 (*h- ade6-M210 leu1–32 ura4-D18*), *Δchk1* *(h- ade6-M210 chk1::ura4+ leu1–32 ura4-D18*), *cdc2.1w* (*h- ade6-M210 leu1–32 ura4-D18 cdc2.1w*), *cdc25.22* *(h- cdc25.22 ade6-M210 leu1–32 ura4-D18*), *Δhhp1* (*h- ade6-M210 hhp1::hphMX6 leu1–32 ura4-D18*), *Δwee1* *(h- ade6-M210 wee1::ura4+ leu1–32 ura4-D18*), Hhp1-HA_3_ (*h- ade6-M210 leu1–32 ura-D18 hhp1::hhp1-HA3-kanMX4*), *Ku70-GFP-HA_3_* (*h90 ade6-M210 leu1–32 lys1–131 ku70::ku70-GFPHA3-kanMX4*), Ku80-HA_3_ (*h90 ade6-M210 leu1–32 ura-D18 ku80::ku80-HA3-ura4+*), Rad16-GFP-HA_3_ (*h90 ade6–216 leu1–32 lys1–131 ura4-D18 rad16::rad16-GFP-HA3-kanMX4*), Srs2-Myc_13_ (*h- ade6–216 leu1–32 ura-D18 srs2::srs2-HA3-kanMX4*), Cdc13-HA_3_ (*h- ade6–216 leu1–32 ura-D18 cdc13::cdc13-HA3-ura4+*), Chk1-HA_3_ (28), Myc_13_-Rqh1 (2), Mus81-Myc_13_ (29), Hhp1-GFP *(hhp1-GFP-kanMX4 ade6–216 leu1–32 ura4-D18*) (30). To construct multiple deletion strains, the following gene deletions were employed: *Δcds1* (*ade6-M210 cds1::ura4+ ura4-D18*), *Δchk1* (*ade6-M210 chk1::kanMX4 leu1–32 ura4-D18*), *Δsrs2* (*ade6-M210 srs2::kanMX4 leu1–32 ura4-D18*), *Δrad3* *(ade6-M210 rad3::ade6+ leu1–32 ura4-D18*), *Δcrb2* (*ade6-M210 crb2::ura4+ leu1–32 ura4-D18*). The *crb2-T215* mutant is described in (18).

### Biochemical techniques

Isoelectric focusing, native protein extracts and total protein extracts are described in (31) and size fractionation is documented in (32).

### Cell synchronisation and DNA repair assays

The preparation of lactose gradients has been reported in (31) and the inter-sister recombination assay is described in (33). The strains used in the recombination tests were: wild type (*h- ade6-M210 leu1–32 ura4-D18 ade6-L469*-*ura4^+^*-*ade6-M375*), *Δsrs2* (*h- ade6-M210 srs2::kanMX4 leu1–32 ura4-D18 ade6-L469-ura4^+^-ade6-M375*), *cdc2.1w* (*cdc2.1w ade6-M210 leu1–32 ura4-D18 ade6-L469-ura4^+^-ade6-M375*) and *cdc2.1w Δsrs2* (*h- cdc2.1w srs2::kanMX4 ade6-M210 leu1–32 ura4-D18 ade6-L469-ura4^+^-ade6-M375*).

Break-induced recombination: The assay uses the *S.cerevisiae* HO (Homothallic switching) endonuclease to cleave the Ch^16^-MG minichromosome at one defined DNA sequence as described in (34). The assay was performed with the following changes: wild-type cells and *cdc2.1w* cells containing Ch^16^-MG were transformed with the plasmid pREP81X-HO-*Leu2+* and maintained on minimal medium plates (+thiamine, + uracil). Single colonies were grown into stationary phase either in the absence or presence of thiamine (15μM thiamine (5ug/ml)) at 30°C in 10 ml of minimal medium (+ uracil). Dilutions were plated on minimal medium plates (+thiamine, +uracil). Colonies were then replica-plated onto rich medium plates with 75 μg/ml G418 to determine the ration of recombinogenic cells. Adenine was omitted at all stages to avoid false-positive colonies which can arise due to the loss of the mini-chromosome.

Plasmid repair assay: cells were transformed with equal amounts of either cut (SacI) or uncut pREP41-*Leu2+* plasmid. Cells were plated on minimal medium plates with uracil and adenine and *leu2^+^* colonies were counted after 4–5 days at 30°C.

### Antibodies

The following antibodies were used: anti-HA (Santa Cruz: SC7392), anti-Myc (Santa Cruz SC40), anti-Cdc2 (Abcam AB70860), anti-GFP (Roche Applied Science 11814460001).

### GFP-Trap

Soluble protein extracts were prepared by breaking 5×10^8^ cells in lysis buffer (50 mM HEPES pH 8.0 200 mM KoAC, 20 mM NaCl, 1 mM EDTA, 0.1% Nonidet, 20 mM beta-glycerol phosphate, 0.1 mM NaF, 1:100 diluted Melford protease inhibitors (IV, P2402), 1 mM DTT). Proteins fused to the Green Fluorescent Protein (GFP) were purified by adding 10 μl of GFP-nAb Agarose (Insight Biotechnology, ABP-NAB-GFPA025) to 1 ml of the lysis buffer containing 100–200 ul soluble protein extract.

## RESULTS

### Elevated Cdc2 activity renders *S. pombe* cells sensitive to CPT

**Figure 1.**
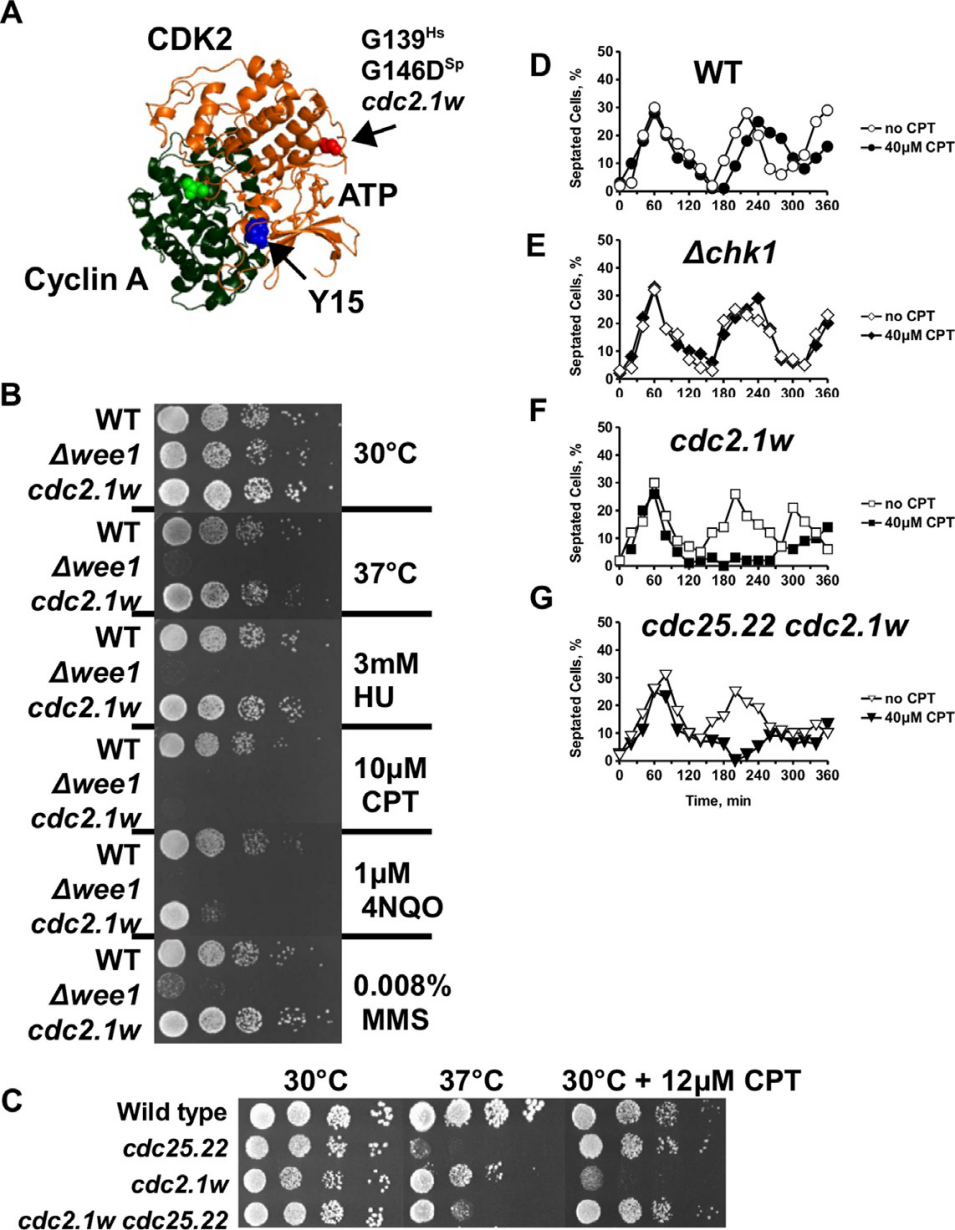
Cells with hyperactive Cdc2.1w kinase are CPT sensitive and enter an extended cell-cycle arrest. **(A)** The G146D mutation in the *S. pombe* Cdc2.1w kinase and the highly conserved Y15 phosphorylation site have been mapped onto the structure of the closely related human CDK2-cyclin A complex (PDB ID: 1FIN) using the program Polyview 3D. **(B)** Drug sensitivity of *cdc2.1w* cells. Serial dilutions (10-fold; starting with 10^7^ cells/ml) of the listed strains were spotted onto YEA plates containing the indicated drugs. The plates were incubated at 30°C for 3 days. One YEA plate was incubated at 37°C. **(C)** Introduction of the *cdc25.22* allele into *cdc2.1w* cells suppresses the CPT sensitivity. **(D–G)** Hyperactive Cdc2.1w causes an extended cell-cycle arrest in the presence of CPT which is not suppressed by the *cdc25.22* allele. The indicated strains were synchronized by lactose gradient centrifugation in early G2 and released into YEA medium with or without 40 μM CPT at 30°C. 40 μl aliquots were withdrawn in 20 min intervals and added to 300 μl methanol. Cells were stained with hoechst (1:1000) and calcofluor (1:100) (calcoflour 1 mg/ml in 50 mM sodium citrate, 100 mM sodium phosphate pH 6.0; hoechst 10 mg/ml in water) prior to scoring under a fluorescence microscope. Open symbols: no CPT, closed symbols: 40 μM CPT.

We took advantage of the *cdc2.1w* (*wee2–1*) mutation in fission yeast to find out whether elevated CDK levels interfere with the DDR. The Cdc2.1w kinase is hyperactive due to a glycine-to-aspartate (G146D) replacement at the entrance to its ATP binding site (Figure 1A). This mutation renders Cdc2.1w insensitive to the inhibition by Wee1 kinase (23,24). Although loss of Wee1 (*Δwee1*) advances entry into mitosis like the *cdc2.1w* mutation, only *Δwee1* cells are known to be DNA damage sensitive (26,27).

Our own survival assays performed with wild type, *Δwee1* and *cdc2.1w* cells confirmed the previous findings for *Δwee1* cells, but also revealed a yet unknown sensitivity of *cdc2.1w* cells to the topoisomerase 1 (Top1) poison camptothecin (CPT) (Figure 1B). CPT immobilises Top1 at the DNA in front of advancing replication forks which break upon their collision with this obstacle (35). To test whether the CPT sensitivity is a consequence of elevated Cdc2 activity, we introduced a temperature-sensitive loss-of-function allele of Cdc25 phosphatase (*cdc25.22*) into the *cdc2.1w* strain. This mutant allele is known to lower Cdc2 activity due to the reduced removal of the inhibitory tyrosine-15 phosphorylation (Figure 1A) (36). As shown in Figure 1C, the *cdc2.1w cdc25.22* double mutant was CPT resistant strongly indicating that high Cdc2 activity interferes with the repair of broken replication forks. We also constructed a *cdc2.1w Δwee1* double mutant to test whether Cdc2.1w and Wee1 act jointly in the response to CPT. Although the *cdc2.1w Δwee1* strain grew more slowly in the presence of the drug, the sensitivity of the double mutant was not increased thus placing both kinases in the same pathway (Supplementary Figure S1A).

### Hyperactive Cdc2 prolongs the G2/M arrest when replication forks break

Yeast cells differ in their response to collapsed replication forks from human cells which arrest in S phase, as they progress through S phase without a delay before briefly (20–40 min) arresting in G2 (37).

We used lactose gradients to synchronze wild type, *Δchk1*, *cdc2.1w* and *cdc2.1w cdc25.22* cells in G2 to analyse their cell-cycle response to 40 μM CPT. Synchronized cells were released into rich medium with and without the drug, and samples were withdrawn every 20 min over 6 h. As shown in Figure 1D, wild-type cells progressed with the same rate through the first G1/S phase (first peak of septation), but delayed entry into the second cycle by 20 min in CPT medium. As expected, this checkpoint response was abolished upon deletion of *chk1* (Figure 1E). Cells with elevated Cdc2 activity showed, however, an unexpected behaviour. They postponed entry into mitosis for 2 h before slowly continuing to cycle (Figure 1F). This extended G2 arrest could be caused by unrepaired replication forks, which continue to send a Rad3^ATR^-Chk1 signal, or by the inability of *cdc2.1w* cells to restart the cell cycle. In contrast to the CPT sensitivity, a reduction in Cdc2.1w activity by the introduction of the *cdc25.22* allele failed to suppress the extended arrest (Figure 1G). This intriguing observation implies that the CPT sensitivity and the prolonged G2/M arrest are two distinct manifestations of the *cdc2.1w* mutation. A possible explanation for the inability of Cdc25.22 to correct the cell-cycle defect lies within mitosis. Although the point mutation lowers the phosphatase activity during interphase, this effect may be neutralised by the 10-fold increase in Cdc25 activity in mitosis (4). If this were to be the case, Cdc2.1w may trigger the cell-cycle defect while cells progress through mitosis, whereas the hyperactive kinase may affect the repair of collapsed forks in G2.

### The G2 arrest in *cdc2.1w* cells is independent of Chk1

**Figure 2.**
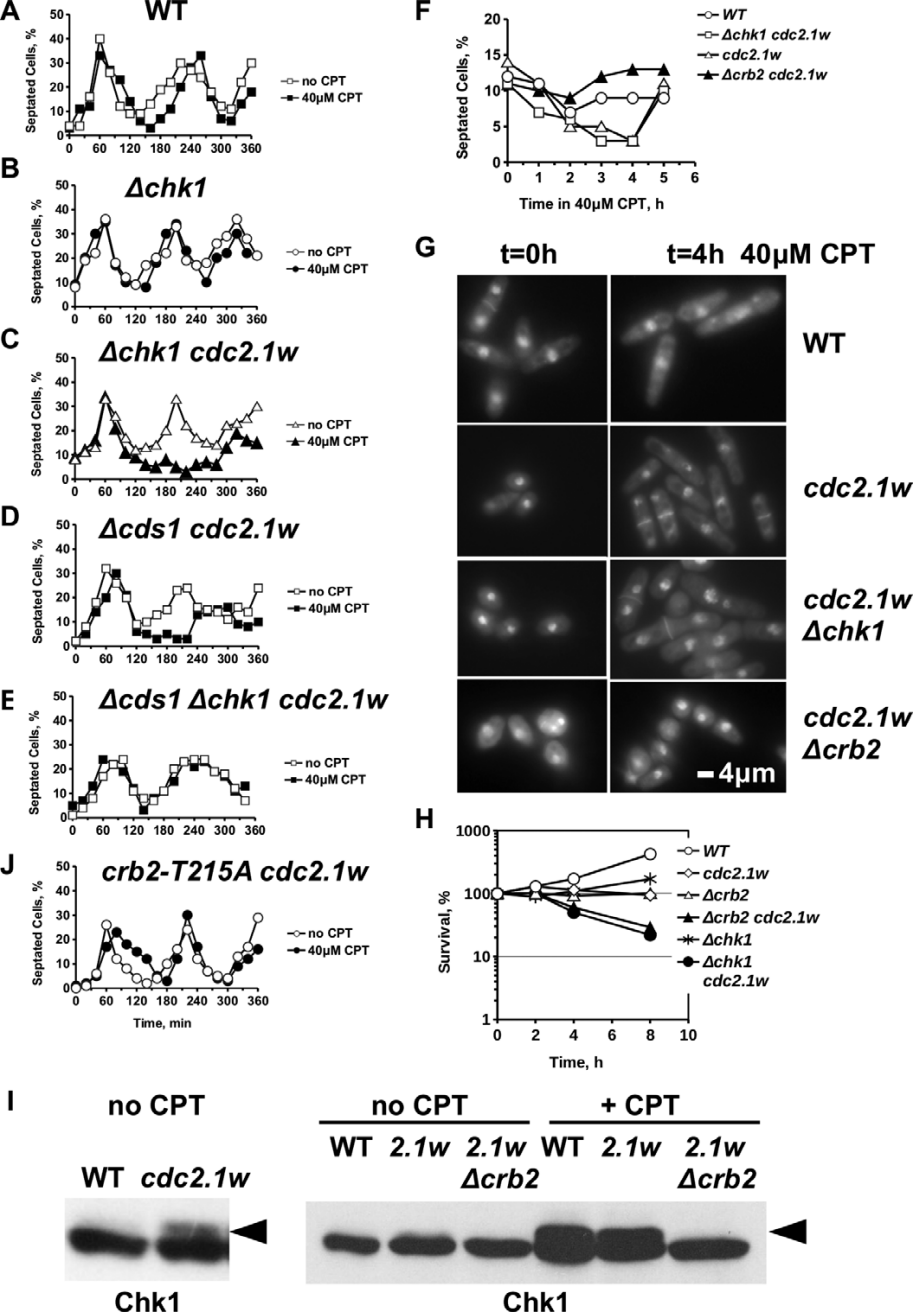
The extended cell-cycle arrest of *cdc2.1w* cells is independent of Chk1 but requires Crb2 and its Cdc2 phosphorylation site T215. **(A–E, J)** Crb2, its phosphorylation site T215 and Cds1 are required for the extended arrest. The indicated strains were synchronized in G2 and released into YEA medium with or without 40 μM CPT at 30°C. Open symbols: no CPT, closed symbols: 40 μM CPT. **(F)** CPT (40 μM) was added to asynchronous cultures of the indicated strains in YEA medium at 30°C and the septation index was analysed over 5 h. **(G)** Loss of *crb2*, but not deletion of *chk1* prevents cell elongation in the presence of CPT. Cells harvested at the start of the experiment (*t* = 0 h) and after 4 h (*t* = 4 h) were inspected under a fluorescence microscope after staining with hoechst (DNA) and calcofluor (septum). **(H)** Cell survival was analysed by incubating 5 × 10^4^ cells/ml of the indicated strains for 8 h in the presence of 40 μM CPT in YEA medium at 30°C. Samples were withdrawn at the indicated time points, plated on YEA plates and incubated for 3 days to analyse viability by colony formation. **(I)** Crb2 is required for the phosphorylation of Chk1 in *cdc2.1w* cells. Chk1-HA cells (WT), Chk1-HA *cdc2.1w* and Chk1-HA *cdc2.1w Δcrb2* cells were left untreated or were incubated for 4 h with 40 μM CPT in YEA medium at 30°C. Total protein extracts were separated on a 10% SDS PAGE and the Chk1 protein was visualized with an anti-HA antibody after western blot. The arrow highlights the slower migrating phospho-band of Chk1. The low levels of phosphorylated Chk1 in undamaged *cdc2.1w* cells as well as the DNA damage induced modification are dependent on Crb2.

Intrigued by this finding, we asked whether hyperactive Cdc2 would also impose a G2/M arrest in *Δchk1* cells given the importance of the Rad3^ATR^-Crb2^53BP1^-Chk1 signalling pathway in the presence of CPT (38). Rather unexpectedly, synchronized *cdc2.1w Δchk1* cells showed a similarly extended G2 arrest in CPT medium as the *cdc2.1w* single mutant (Figure 2C). This shows that hyperactive Cdc2 bypasses Chk1 to block entry into mitosis when replication forks break. Since Rad3 kinase signals either through Chk1 or Cds1^Chk2^ (39), we also tested a *cdc2.1w Δcds1* double mutant. Elevated Cdc2 activity did also impose a G2 arrest in the absence of Cds1 but this arrest was ∼1 h shorter compared to the *cdc2.1w Δchk1* mutant (Figure 2D). The ability of the *cdc2.1w Δcds1* mutant to arrest and the observation that Chk1 is not required in *cdc2.1w* cells (Figure 2C) could be explained in two ways. Either a third unknown kinase is involved, or Chk1 is not important in *cdc2.1w* cells as long as Cds1 is intact. To distinguish between these possibilities, we measured the cell-cycle arrest of a *Δchk1 Δcds1 cdc2.1w* triple mutant in CPT medium. Interestingly, loss of both kinases completely abolished the G2/M arrest (Figure 2E) showing that Cds1 acts first in *cdc2.1w* cells when forks break rendering Chk1 redundant. This is an intriguing observation since Chk1 is normally the key kinase in the presence of CPT (38).

### The G2 arrest in *cdc2.1w* cells requires the CDK phosphorylation site T215 in Crb2

Although *cdc2.1w* cells bypass Chk1, we still wanted to test the requirement of the Chk1-adaptor protein Crb2^53BP1,Rad9^ given that Cdc2 phosphorylates Crb2 at T215 in mitosis (18). Deletion of *crb2* in *cdc2.1w* cells resulted in very short cells which were difficult to synchronise (Figure 2G). To circumvent this problem, we added 40 μM CPT directly to asynchronous cultures of wild type, *cdc2.1w*, *Δchk1 cdc2.1w* and *Δcrb2 cdc2.1w* cells and followed the septation index over 5 h. As shown in Figure 2F, the septation index of *cdc2.1w* and *Δchk1 cdc2.1w* cells dropped after the first hour to 2% and started to rise again after 4 h. This decline in dividing cells is consistent with an extended G2/M arrest. We also took samples at the start of the experiment (*t* = 0) and after 4 h to examine cells under the microscope. Fission yeast cells which stop in G2 continue to grow and become elongated (27). While *cdc2.1w* and *cdc2.1w Δchk1* cells clearly elongated in CPT medium, *cdc2.1w Δcrb2* cells maintained their short cell size (Figure 2G). Consistent with the absence of cell elongation, the septation index of this double mutant failed to drop (Figure 2F). These findings show that Crb2 is required for the arrest in *cdc2.1w* cells despite the independence on Chk1. Since *cdc2.1w Δcrb2* and *cdc2.1w Δchk1* cells lost their viability to a similar extent in the presence of CPT (Figure 2H), the ability to postpone mitosis is not linked with enhanced cell survival.

To identify Crb2 but not Chk1 as a component of the extended G2 arrest in *cdc2.1w* cells was a surprise especially since our earlier observations implicated Cds1 kinase which is normally activated by Mrc1 and not by Crb2 (40). The requirement for Crb2 left us, however, with a conundrum since we and others have found that Chk1 is phosphorylated in undamaged cells with hyperactive Cdc2 as well as in the presence of CPT (Figure 2I) (41,31). Consistent with the role of Crb2 as a Chk1 adaptor, the band shift caused by the phosphorylation of Chk1 at S345 in response to CPT was abolished in *cdc2.1w Δcrb2 chk1-HA* cells (Figure 2I). An explanation of why Chk1 is modified in a Crb2-dependent manner in *cdc2.1w* cells in the presence of DNA damage despite its unimportance for the mitotic arrest could be provided by the two independent modes of Crb2 recruitment to DNA. Phosphorylation of Crb2 at T215 by Cdc2 in mitosis directs the protein to undamaged DNA (18), whereas its T215-independent interactions with methylated and phosphorylated histones direct Crb2 to damaged DNA in G2 (20). Hence, hyperactive Cdc2.1w may only affect the T215 phosphorylated pool of Crb2, but not the DNA damage activated pool. Consistent with this notion, replacement of T215 by an alanine residue (T215A) prevented *crb2-T215A cdc2.1w* cells from arresting the cell cycle in CPT medium (Figure 2J). This important finding suggests that the mitotic modification of Crb2-T215 by Cdc2 may interfere with the activation of Chk1 when replication forks break in *cdc2.1w* cells.

### Elevated Cdc2 activity locks cells in a G1-like DNA repair state

**Figure 3.**
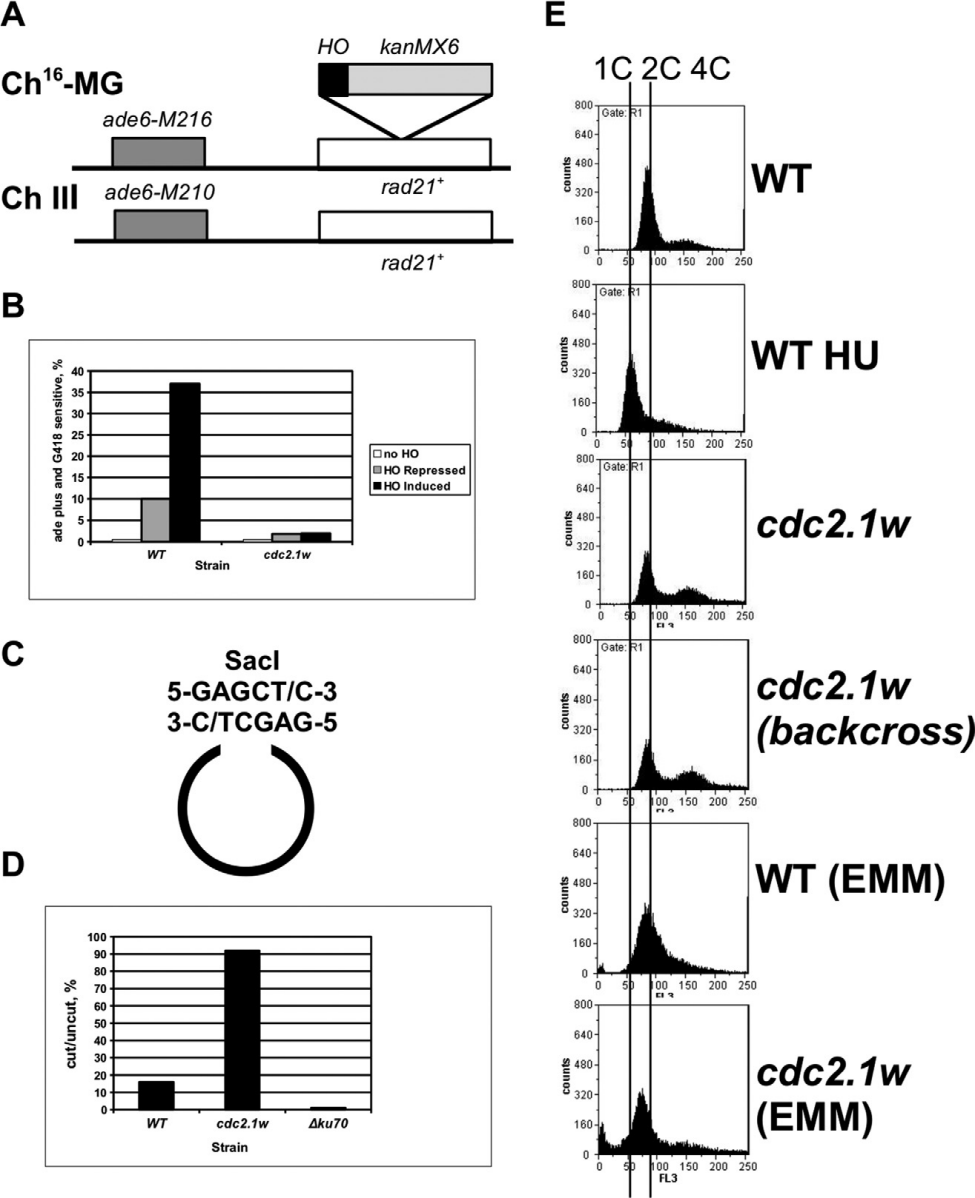
Elevated Cdc2 activity locks cells in a G1-like DNA repair mode. **(A)** Principle of the Ch^16^-MG Assay. **(B)** Wild-type cells and *cdc2.1w* cells containing the mini-chromosome Ch^16^-MG were grown in the absence of the HO endonuclease (No HO, pREP41 plasmid) or the presence of the HO enzyme either with (HO Repressed) or without thiamine (HO Induced). The averages of *ade6+ G418S* colonies from three independent experiments are shown. **(C)** Principle of the plasmid repair assay. **(D)** The average ratios of tranformants for the linearized vector normalized against the uncut plasmid from three independent experiments are shown. **(E)** The DNA content was measured using flow cytometry from asynchronous wild type and *cdc2.1w* cultures grown in rich medium or in minimal medium (EMM). Wild-type cells were arrested at the G1/S boundary with 12 mM HU for 4 h to have an internal standard for a 1C DNA content (WT HU). To exclude the presence of genetic alterations in the *cdc2.1w* strain, the strain was back-crossed against wild-type strains and the analysis was repeated (backcross).

Given the importance of Cdc2^Cdc28^ activity for the recombinogenic repair of broken chromosomes in *S.cerevisiae* (1), we wanted to test whether elevated recombination levels interfere with the repair of collapsed replication forks in *cdc2.1w* cells. To test this idea, we measured break-induced HR by using a genetic system which allows for the genetic exchange between chromosome III and the homologous mini-chromosome Ch^16^-MG upon its cleavage by HO endonuclease (Figure 3A) (34). Prior to HO expression from the inducible *nmt81* promotor (pREP81X-HO), cells containing Ch^16^-MG grow in the absence of adenine (ade6^+^) and in the presence of the antibiotic G418 (G418^R^). Following HO induction, the cleavage of Ch^16^-MG at its unique HO site within the engineered *rad21* gene will trigger DNA repair. While homologous recombination between the two *rad21* genes will result in the loss of the G418 resistance cassette due to DNA end resection (ade6^+^ G418^S^), NHEJ of the HO break will retain the antibiotic resistance (ade6^+^ G418^R^). Approximately 30%–40% of wild-type cells grown in minimal medium without thiamine (repressor of the *nmt81* promotor) underwent recombination, whereas only ∼10% were recombinogenic in the presence of the repressor (Figure 3B). The latter is due to the leaky nature of the *nmt81* promotor. Unexpectedly, less than 2% of *cdc2.1w* cells underwent recombination in thiamine-free medium (Figure 3B). This intriguing finding suggests that elevated Cdc2.1w activity favours NHEJ over interchromosomal recombination as previously reported for *S. pombe* cells arrested in G1 (3). While this observation contradicts the importance of Cdc2^Cdc28^ activity for DNA end resection as observed in *S.cerevisiae* (1), it is consistent with a recent report showing that an aberrant increase in Cdc2 activity blocks interchromosomal recombination in human cells (42).

Since this finding suggests an increase in NHEJ in *cdc2.1w* cells, we measured NHEJ using a plasmid repair assay (3). The plasmid pREP41 was linearized at its unique SacI restriction site (Figure 3C) and equal amounts of cut and uncut plasmid were transformed into asynchronous wild type, *cdc2.1w* and NHEJ-deficient *Δku70* cells. While less than 20% of wild type cells were able to repair the plasmid, more than 90% of *cdc2.1w* cells were proficient in this assay (Figure 3D). As G1 cells utilize NHEJ over HR (3), we analysed the DNA content of asynchronous wild type and *cdc2.1w* cultures grown in rich and minimal medium using flow cytometry (43). Although G2 is ∼20 min shorter in *cdc2.1w* cells than in wild type cells (Figure 1F, Supplementary Figure S1B–E), we did not detect an increase in G1 cells in *cdc2.1w* cultures independently of the growth medium (Figure 3E). Taken together, these experiments support the conclusion that elevated Cdc2 activity locks *S.pombe* cells in a G1-like DNA repair state which may compromise the recovery of collapsed replication forks.

### Cdc2 associates with Srs2 DNA helicase, Hhp1 kinase, Chk1 kinase and the Ku70-Ku80 DNA binding complex

**Figure 4.**
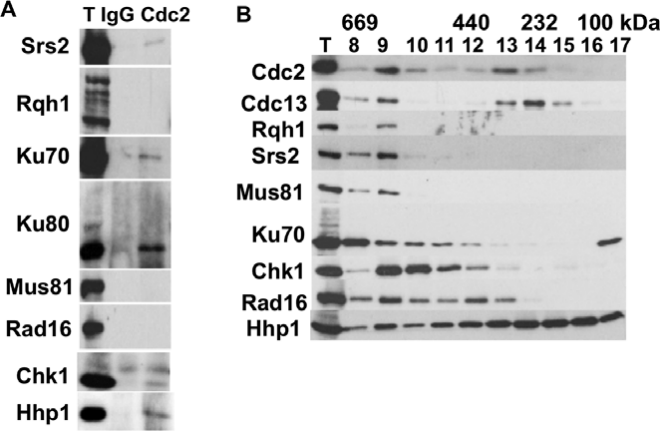
Cdc2 associates with Srs2, Ku70, Ku80, Hhp1 and Chk1. **(A)** Native protein extracts (T) (150 μl) prepared from untreated cells were incubated with 5 μl of an unrelated IgG antibody or 5 μl of an anti-Cdc2 antibody over night. Protein-antibody complexes were harvested from the supernatant by the addition of 30 μl protein A/G beads (Calbiochem) and analysed using an affinity tag-specific antibody (Srs2-Myc, Myc-Rqh1, Ku70-GFP-HA, Ku80-HA, Mus81-Myc, Rad16-GFP-HA, Chk1-HA, Hhp1-HA). **(B)** Size fractionation of the affinity tagged strains on a Superdex-200 column. (Total = native protein extract). The fractions obtained from the Cdc13-HA extract were also probed with an anti-Cdc2 antibody.

To find out how Cdc2.1w affects Crb2 and the DNA repair status of cells, we performed a small-scale immunoprecipitation screen to identify DDR proteins which bind to the kinase. Soluble extracts prepared from undamaged strains expressing affinity-tagged versions of Srs2 DNA helicase, Rqh1^BLM^ DNA helicase, Ku70, Ku80, Mus81 endonuclease, Rad16^XPF^, Chk1 kinase or Casein kinase 1 (Hhp1) were incubated with an anti-Cdc2 antibody and an unrelated immunoglobulin G (IgG) antibody and then precipitated with protein A/G beads. As shown in Figure 4A, small amounts of Ku70-Ku80, Srs2, Chk1 and Hhp1 were pulled down with the anti-Cdc2 antibody identifying these proteins as potential binding partners. Casein Kinase 1 was included in this experiment because we noted a strong genetic interaction between a loss-of-function *wee1* mutation (*wee1–50*) and the deletion of *hhp1* in an independent experiment (Figure 6A).

**Figure 5.**
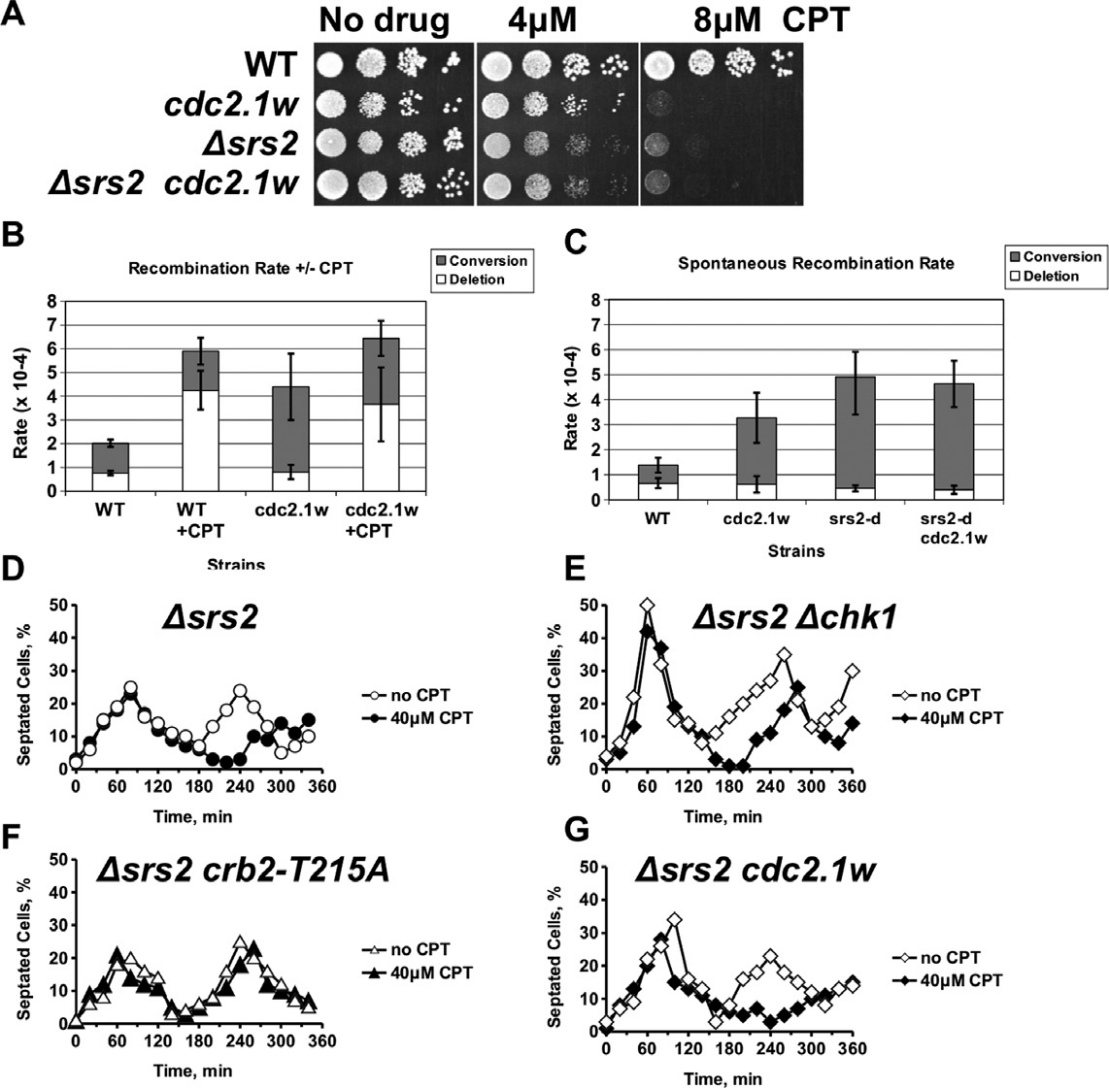
Hyperactive Cdc2.1w blocks Srs2 DNA helicase and loss of *srs2* arrests cell-cycle progression in a Crb2-T215 dependent manner. **(A)** Cdc2.1w and Srs2 act in the same CPT response pathway. **(B)** CPT induces deletion type recombination events. Wild type and *cdc2.1w* cells containing the recombination cassette (*ade6-L469*-*ura4^+^*-*ade6-M375*) were grown in 5 ml YEA medium with or without 10 μM CPT from a single colony into stationary phase. Cell dilutions were plated onto minimal medium plates (100 mg/L uracil, 100 mg/L leucine, 200 mg/L guanine) to select for cells with a restored *ade6* gene (= recombination event). Loss of the *ura4* marker (= deletion event) was determined by replica-plating onto minimal medium plates (100 mg/L leucine, 200 mg/L guanine). Open boxes = deletion events, closed boxes = conversion events. **(C)** Cdc2.1w increases spontaneous gene conversion events in a Srs2-dependent manner. Strains of the indicated genotypes were grown in YEA medium without drug into stationary phase and analysed. **(D–G)** Loss of Srs2 delays cell-cycle progression in the presence of CPT independently of Chk1, but dependent on the Cdc2 phosphorylation site Crb2-T215. Cells of the indicated genotypes were synchronized in G2 and released in YEA medium with or without 40 μM CPT at 30°C. Open symbols: no CPT, closed symbols: 40 μM CPT.

**Figure 6.**
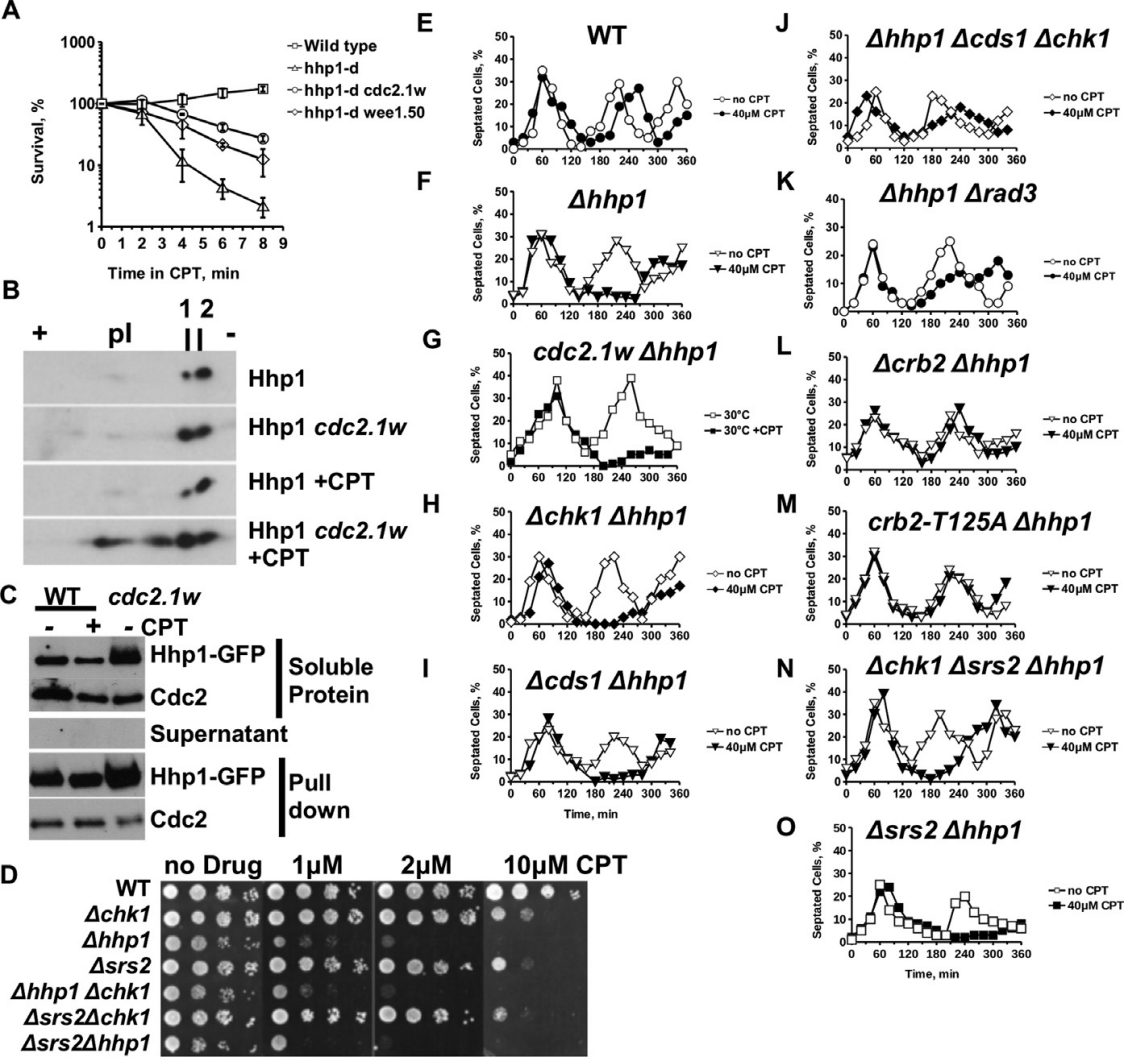
Cdc2 targets Casein kinase 1 (Hhp1) and loss of Hhp1 delays the cell cycle independently of Chk1 but requires Cds1 and the Cdc2 phosphorylation site Crb2-T215. **(A)** Hyperactive Cdc2.1w partly suppresses the CPT sensitivity of *Δhhp1* cells (40 μM CPT in YEA medium at 30°C). **(B)** Cdc2 targets Hhp1 kinase. Isoelectric focusing of total protein extracts prepared from Hhp1-HA and Hhp1-HA *cdc2.1w* cells either treated with 40 μM CPT at 30°C for 4 h or left untreated. **(C)** Hhp1-GFP was purified from wild-type cells in the absence or presence of CPT (40 μM, 4 h) and from untreated Hhp1-GFP *cdc2.1w* cells using the GFP-trap. Samples of the total soluble extracts and of the purified material were probed with an anti-GFP and an anti-Cdc2 antibody. Samples of the supernatant after the pull-down were probed with the anti-GFP antibody. **(D)** Hhp1 kinase acts in the same CPT response as Srs2 and Chk1. **(E–O)** Loss of Hhp1 delays cell-cycle progression in the presence of CPT independently of Chk1, but dependent on the Cdc2 phosphorylation site Crb2-T215, Rad3 and Cds1. Cells of the indicated genotypes were synchronized in G2 and released in YEA medium with or without 40 μM CPT 30°C. Open symbols: no CPT, closed symbols: 40 μM CPT.

Size fractionation of soluble extracts obtained from these strains revealed two peaks of Cdc2 and cyclin B (Cdc13) at 600 kDa and 250 kDa, respectively (Figure 4B). All potential Cdc2 binding partners coeluted with Cdc2 in the higher molecular weight range of 600 kDa suggesting that they form larger protein complexes. Interestingly, monomeric protein was only detected for Ku70 and Hhp1, whereas all other proteins eluted with an apparent molecular weight well above their expected sizes.

For the rest of the project, we focused on Srs2 and Hhp1 since deletion of either gene was epistatic with the *cdc2.1w* mutation. The biology of the interaction between Ku70–Ku80 and Cdc2 will be reported somewhere else.

### Loss of Srs2 DNA helicase prolongs the CPT-induced G2 arrest in a *crb2-T215A* dependent manner

Srs2 is a multifunctional DNA helicase which helps to join DNA ends with microhomologies during NHEJ (44), prevents unwanted HR by dismantling Rad51-ssDNA filaments (45), promotes HR by resolving D-loop structures during strand invasion (46) and binds to different replication structures *in vitro* (47). These opposing activities are regulated by CDK1 in *S.cerevisiae* which phosphorylates Srs2 at multiple sites to stimulate HR in S/G2 (46).

Consistent with the association of Cdc2 with Srs2 (Figure 4A), we found an epistatic relationship between a *srs2* deletion and the *cdc2.1w* allele for the survival in the presence of CPT (Figure 5A). Since loss of *srs2* increases the spontaneous exchange between sister chromatids (48), we measured inter*-*sister recombination rates by employing an assay which monitors the restoration of adenine independence upon the recombination between two tandem *ade6-negative* heteroalleles which are separated by a functional *ura4^+^* marker (*ade6-L469-ura4^+^-ade6-M375*) (33). The *ura4^+^* gene enabled us to distinguish *ade6^+^* recombinants that had lost (deletion type) or retained (conversion type) the intervening DNA between the *ade6* repeats. Deletion of the *ura4^+^* marker is indicative of recombination at collapsed forks (49), whereas spontaneous recombination between sister chromatids in G2 retains the intervening sequence (conversion type). As shown in Figure 5B, wild type and *cdc2.1w* cells both suffered from a 5-fold increase in gene deletions in the presence of 10 μM CPT (WT: no drug: 0.8 × 10^−4^; CPT: 4.3 × 10^−4^; *cdc2.1w*: no drug: 0.8 × 10^−4^; CPT: 3.7 × 10^−4^). This finding confirms the recombinogenic nature of collapsed forks, but also shows that uncontrolled genetic exchange at these structures is not the cause of death in *cdc2.1w* cells. Although recombination at broken forks is normal in *cdc2.1w* cells, the spontaneous exchange between sister chromatids in undamaged cells was 3-fold higher than in wild-type cells (WT: 1.3 × 10^−4^; *cdc2.1w*: 3.6 × 10^−4^) (Figure 5B). Since such unwanted recombination is prevented by Srs2 (48), we measured the conversion rates in undamaged wild type, *cdc2.1w*, *Δsrs2* and *Δsrs2 cdc2.1w* cells. Cells without Srs2 had a 6-fold higher rate compared to wild type, which was not further increased in the *Δsrs2 cdc2.1w* double mutant (WT: 0.7 × 10^−4^; *cdc2.1w*: 2.7 × 10^−4^; *Δsrs2*: 4.5 × 10^−4^; *cdc2.1w Δsrs2*: 4.3 × 10^−4^) (Figure 5C). In summary, these results imply that hyperactive Cdc2 has two effects on Srs2. Its anti-recombination activity is down-regulated in undamaged cells leading to elevated rates of spontaneous inter-sister exchange and also its DNA repair function is blocked when replication forks collapse resulting in CPT sensitivity. This close functional relationship between Cdc2 and Srs2 implies that the kinase modifies Srs2. Although *S.cerevisiae* Srs2 is phosphoryated by Cdc2^Cdc28^ (46), a similar modification has not yet been reported in *S. pombe*.

Given the close functional link between Cdc2 and Srs2, we measured the G2 arrest in synchronized *Δsrs2*, *Δsrs2 Δchk1*, *Δsrs2 cdc2.1w* and *Δsrs2 crb2-T215A* cells. Intriguingly, loss of the helicase resembled the hyperactive *cdc2.1w* mutation because the extended G2 arrest of *Δsrs2* cells was independent of Chk1 (Figure 5E) but required the phosphorylation of Crb2 at T215 (Figure 5F). One important difference between *cdc2.1w* and *Δsrs2* cells was, however, the shorter G2 arrest when forks collapsed in the absence of the DNA helicase. While *cdc2.1w* cells delayed entry into mitosis for up to 2 h (Figure 1F), *Δsrs2* cells delayed only for 40–60 min (Figure 5D).

In summary, these findings imply that Cdc2 kinase targets Srs2 in *S.pombe* and that the hyperactive kinase may switch Srs2 activity from the prevention of spontaneous inter*-*sister recombination to the promotion of NHEJ. They also show that elevated Cdc2 activity has distinct effects on HR. While break-induced recombination between homologous chromosomes is blocked (Figure 3B), sponatenous recombination between sister chromatids, which are attached by the cohesion complexes, is increased (Figure 5B and C).

### Casein kinase 1 (Hhp1) is aberrantly modified in *cdc2.1w* cells

We became interested in CK1 because the CPT sensitivity of the *Δhhp1* deletion strain was partly suppressed by elevated Cdc2 activity (*cdc2.1w* or *wee1–50*) (Figure 6A). This suppression was, however, limited to the acute exposure of cells to the topoisomerase 1 inhibitor as it was not evident when cells were grown for several days in the presence of the drug. This rescue places both kinases in the same CPT response, a conclusion supported by their physical association (Figure 4A). Since Hhp1 is known to undergo autophosphorylation (50), we employed isoelectric focusing to investigate the modification pattern of the kinase in wild type and *cdc2.1w* cells treated with 40 μM CPT for 4 h or left untreated. Separation of soluble Hhp1-HA protein on a linear pH strip ranging from pH3 to pH10 revealed two species of CK1 (Figure 6B). The intensity of the more acidic form (number 1 in Figure 6B) increased in *cdc2.1w hhp1-HA* cells independently of CPT. This observation, together with the direct association between the two kinases (Figure 4A), implies that Cdc2 phosphorylates Hhp1.

While Hhp1 was not further modified in CPT-treated wild-type cells, several hypermodified species appeared when *cdc2.1w hhp1-HA* were incubated with the drug (Figure 6B, panel 4). These additional modifications could arise from aberrant Cdc2 activity in the presence of CPT, from a change in autophosphorylation of Hhp1 in *cdc2.1w* cells or from a yet unknown kinase targeting Hhp1 under these conditions.

To test whether the association of Cdc2 with Hhp1 is affected by CPT treatment or elevated Cdc2 activity, we purified Hhp1-GFP protein complexes from growing cells, cells treated for 4 h with 40 μM CPT or untreated *cdc2.1w* Hhp1-GFP cells using the novel GFP-trap. As shown in Figure 6C, the high affinity GFP-binding protein depleted the soluble Hhp1-GFP protein from the extract. The purified pool of Hhp1-GFP contained a significant amount of Cdc2 kinase independently of CPT treatment and high Cdc2 activity suggesting a stable interaction between the kinases.

Informed by the previous finding that hyperactive Cdc2 down-regulates Srs2 (Figure 5), we tested the CPT sensitivity of *Δsrs2 Δhhp1* cells and found an epistatic relationship between CK1 and the helicase. We also found an epistatic relationship between Chk1 and Srs2, and between Chk1 and Hhp1 (Figure 6D). Taken together, these observations imply that Hhp1 and Srs2 both have to be active for Chk1 to become stimulated by broken replication forks.

### Deletion of Casein Kinase 1 prolongs the G2 arrest in a *crb2-T215A* dependent manner

To test whether Hhp1 acts in the G2 arrest of *cdc2.1w* cells, we synchronized wild type, *Δhhp1* and *cdc2.1w Δhhp1* cells and measured cell-cycle progression in the presence of 40 μM CPT. Interestingly, deletion of *hhp1* on its own was sufficient to delay entry into mitosis by ∼2 h (Figure 6F) in a Chk1-independent manner (Figure 6H). In light of the association of Hhp1 with Cdc2 (Figure 4A) and the Cdc2.1w-dependent change in its phosphorylation pattern (Figure 6B), it seems very likely that hyperactive Cdc2 alters Crb2 activities by blocking Hhp1 kinase. Consistent with this conclusion, loss of *crb2* (Figure 6L) or mutation of T215 to alanine (Figure 6M) abolished the G2 arrest of *Δhhp1* cells in the presence of CPT.

Since our earlier data on *cdc2.1w* strongly suggest that the mitotic Crb2-T215 modification allows Rad3 to activate Cds1 instead of Chk1 (Figure 2E), we deleted *cds1*, *rad3* or *cds1* and *chk1* in the *Δhhp1* mutant. While loss of Cds1 on its own had no effect on the arrest (Figure 6I), concomitant deletion of *cds1* and *chk1* or deletion of *rad3* abolished the G2 delay (Figure 6J and K). These observations are in agreement with our previous finding that *Δcds1 Δchk1 cdc2.1w* cells fail to stop in CPT medium (Figure 2E) and show that Chk1 becomes only important in *Δhhp1* cells when Cds1 is inactivated. In line with the epistatic relationship between Hhp1 and Srs2 (Figure 6D), loss of Srs2 in *Δhhp1* or in *Δhhp1Δchk1* cells had no further effect on the arrest although the *Δhhp1Δchk1Δsrs2* triple mutant reentered the cell cycle earlier compared to the *Δhhp1Δsrs2* double mutant (Figure 6N and O).

## DISCUSSION

**Figure 7.**
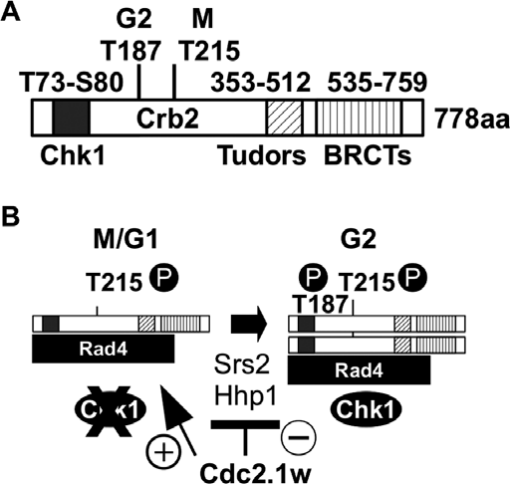
Model. **(A)** The domain structure of Crb2. In the presence of DNA damage, the C-terminal Tudor and BRCT domains allow Crb2 to bind to methylated and phosphoryalated histones, respectively. Cdc2 phosphorylates Crb2 at threonine 215 (T215) in mitosis thereby promoting the association of Crb2 with Rad4 independently of DNA damage. Chk1 associates with Crb2 after the Rad3-dependent phosphorylation of T73 and S80 in the response to DNA lesions once Crb2 has been modified at T187 by Cdc2 in G2. **(B)** Model. In wild-type cells, the Crb2-Rad4 complex changes from its M/G1 configuration to its G2 configuration when cells exit S phase. This transition is promoted by Srs2 DNA helicase and Hhp1 (CK1) kinase, and by the G2 modification of T187 by Cdc2. In *cdc2.1w* cells, this transition is delayed due to the inhibition of Srs2 and Hhp1 by the hyperactive Cdc2.1w kinase. This locks the Crb2-Rad4 complex in its M/G1 mode. The repair of broken replication forks may be delayed as *cdc2.1w* cells favour NHEJ over interchromosomal recombination. This leads to the activation of Cds1 instead of Chk1, and an extended G2 arrest.

We report here that fission yeast cells with a hyperactive Cdc2 kinase (*cdc2.1w*) are specifically sensitive to the topoisomerase 1 inhibitor CPT (Figure 1B), enter a prolonged G2 arrest when replication forks break in the presence of CPT (Figure 1F) and maintain a G1-like DNA repair state with high levels of NHEJ and low levels of interchromosomal recombination (Figure 3). Our genetic data strongly suggest that the CPT sensitivity and the extended G2 arrest are two independent manifestations of elevated Cdc2 activity. While the introduction of a loss-of-function mutation in Cdc25 phosphatase (*cdc25.22*), which is known to lower Cdc2.1w activity (24), suppresses the CPT sensitivity (Figure 1C), it fails to restore a normal G2/M delay (Figure 1G). This difference could be explained by the dynamics of Cdc2 throughout the cell cycle. *In vitro* kinase assays have shown that Cdc2 activity starts to increase half way through G2 in normal fission yeast cells, but starts very early in G2 and then rises at twice the rate in cells with hyperactive Cdc2 kinase (51). This change in Cdc2 levels throughout the cell cycle could have a profound effect on Crb2 as Cdc2 targets the protein at least twice in one cycle. At its activity peak in mitosis, Cdc2 phosphorylates Crb2 at T215 which allows Crb2 to bind to Rad4 (18,19). The mitotic Crb2-Rad4 complex may exist until the start of G2 when sufficient Cdc2 activity accumulated again to modify the complex at T187, a non-canonical Cdc2 site closer to the N-terminus of Crb2 (Figure 7) (21). The phosphorylation of T187 rearranges the Crb2-Rad4 complex so that Crb2 can bind to Chk1, and Rad4 to the Rad9-Rad1-Hus1 complex (21). This temporal order of modifications may be affected by the higher and faster rising levels of Cdc2 in *cdc2.1w* cells as they fail to activate Chk1 when replication forks collapse (Figure 2C). Intriguingly, *cdc2.1w* cells activate Cds1 kinase under these conditions (Figure 2D and E) which implies that the mitotic Crb2-Rad4 complex targets this kinase instead of Chk1. Elevated Cdc2 activity may either expand the mitotic pool of the T215 phosphorylated Crb2-Rad4 complex or it may initiate further modifications like the phosphorylation of T187 prematurely. As a result of this, the Crb2-Rad4 complex may become trapped in its mitotic state (52) thereby promoting NHEJ over interchromosomal recombination (Figure 3). If replication forks were to break under these conditions, their repair may be delayed explaining the extended G2 arrest (Figure 7). Interestingly, the aberrant modification of the Crb2-Rad4 complex in *cdc2.1w* cells requires the inhibition of Hhp1 kinase and Srs2 DNA helicase as *Δhhp1* and *Δsrs2* mutants both show an extended G2 arrest which is abolished upon mutation of T215 (Figures 5F and 6M). Since Hhp1 and Srs2 associate both with Cdc2 (Figure 4), the hyperactive kinase may either block or modulate their activities to promote NHEJ and/or to expand the pool of T215 modified Crb2-Rad4 complexes.

Budding yeast Srs2 possesses several functions which may help to explain how a multifunctional helicase could regulate Crb2. Srs2^Sc^ binds *in vitro* to junctions between single- and double-stranded DNA (47) and promotes NHEJ in G1 (44). If Crb2, like human 53BP1, prevents the resection of DNA ends in G1, Srs2 may terminate this activity by binding to these junctions at the G1/S transition. In normal cells, Srs2 may therefore be down-regulated by Cdc2 until cells initiate DNA replication. This inhibition may well be extended beyond the start of S phase in *cdc2.1w* cells causing problems with the activation of the DNA damage checkpoint kinases when forks break. Although hyperactive Cdc2.1w clearly blocks Srs2 as indicated by the increase in the spontaneous inter-sister recombination rate (Figure 5C), it is as yet unclear whether this is by a Cdc2-dependent modification of the helicase. The latter is, however, supported by the physical association between Cdc2 and Srs2 (Figure 4A), and by similar findings in *S.cerevisiae* (46). Alternatively, Cdc2 could regulate Srs2 indirectly via Hhp1 given the epistatic relationship between *Δsrs2* and *Δhhp1* for the CPT sensitivity (Figure 6D) and the G2 arrest (Figure 6O).

Hhp1 is most closely related to human Casein kinase 1ϵ (53), which performs diverse roles in the circadian clock, DNA repair and wnt-β-catenin signaling (54). CK1 enzymes are monomeric kinases which are regulated by autophosphorylation and require in many cases a priming kinase to recognize a substrate (54). There are several ways how Hhp1 could regulate Crb2. Hhp1 could act through Srs2, but this is less likely since the duration of the G2 arrest is significantly longer in *Δhhp1* cells (Figure 6F) than in *Δsrs2* cells (Figure 5D). Alternatively, Srs2 may associate with and regulate Hhp1. This idea is based on the recent discovery that human CK1ϵ associates with the RNA helicase DDX3 to control wnt-β-catenin signalling (55). A third possibility is that Cdc2 primes Crb2 for the phosphorylation by Hhp1 and that this modification is required for Srs2 to act on Crb2. In line with this notion, additional phosphorylation bands were observed once Crb2 was modified by Cdc2 at T215 (18). Whether this hyperphosphorylation of Crb2 is dependent on CK1 is not yet clear, but the *S.cerevisiae* paralog of Crb2, Rad9, is modified by Polo-like kinase and Casein kinase 2 when cells reenter the cell cycle from a G2 arrest (56).

In summary, our data entertain a model (Figure 7) in which Cdc2 retains the Crb2-Rad4 complex in its M/G1 mode by blocking Srs2 DNA helicase and Hhp1 kinase until cells enter G2 phase. This would silence Chk1 until sufficient Cdc2 kinase has accumulate at the start of G2, which may be a prerequisite to promote NHEJ in G1 in wild-type cells. In *cdc2.1w* cells, this inhibition of Chk1 seems to continue beyond the start of G2 resulting in the aberrant activation of Cds1 and a long G2 arrest when forks break in a chromatin environment that favours NHEJ over interchromosomal recombination. Further work is, however, required to establish how Cds1 is activated in *cdc2.1w* cells and how Hhp1 and Srs2 are regulated by Cdc2 to modulate Crb2.

## SUPPLEMENTARY DATA

Supplementary Data are available at NAR Online

SUPPORTING INFORMATION
