# Supplementary material for: Hyperactive Cdc2 kinase interferes with the response to broken replication forks by trapping S.pombe Crb2 in its mitotic T215 phosphorylated state
Source: Nucleic Acids Res. 2014 May 26;42(12):7734–47. doi: 10.1093/nar/gku452 (PMC4081076; doi:10.1093/nar/gku452)
Supplement: SUPPORTING INFORMATION [file supp_42_12_7734__index.html]

Hyperactive Cdc2 kinase interferes with the response to broken replication forks by trapping S.pombe Crb2 in its mitotic T215 phosphorylated state — Hyperactive Cdc2 kinase interferes with the response to broken replication forks by trapping S.pombe Crb2 in its mitotic T215 phosphorylated state — SUPPORTING INFORMATION 

# Hyperactive Cdc2 kinase interferes with the response to broken replication forks by trapping *S.pombe* Crb2 in its mitotic T215 phosphorylated state

## SUPPORTING INFORMATION

**Files in this Data Supplement:**

- Supplemental Figures
