# Supplementary material for: Hyperactive Cdc2 kinase interferes with the response to broken replication forks by trapping S.pombe Crb2 in its mitotic T215 phosphorylated state
Source: Nucleic Acids Res. 2014 May 26;42(12):7734–47. doi: 10.1093/nar/gku452 (PMC4081076; doi:10.1093/nar/gku452)
Supplement: SUPPORTING INFORMATION [file supp_gku452_nar-03133-d-2013-File009.pdf]

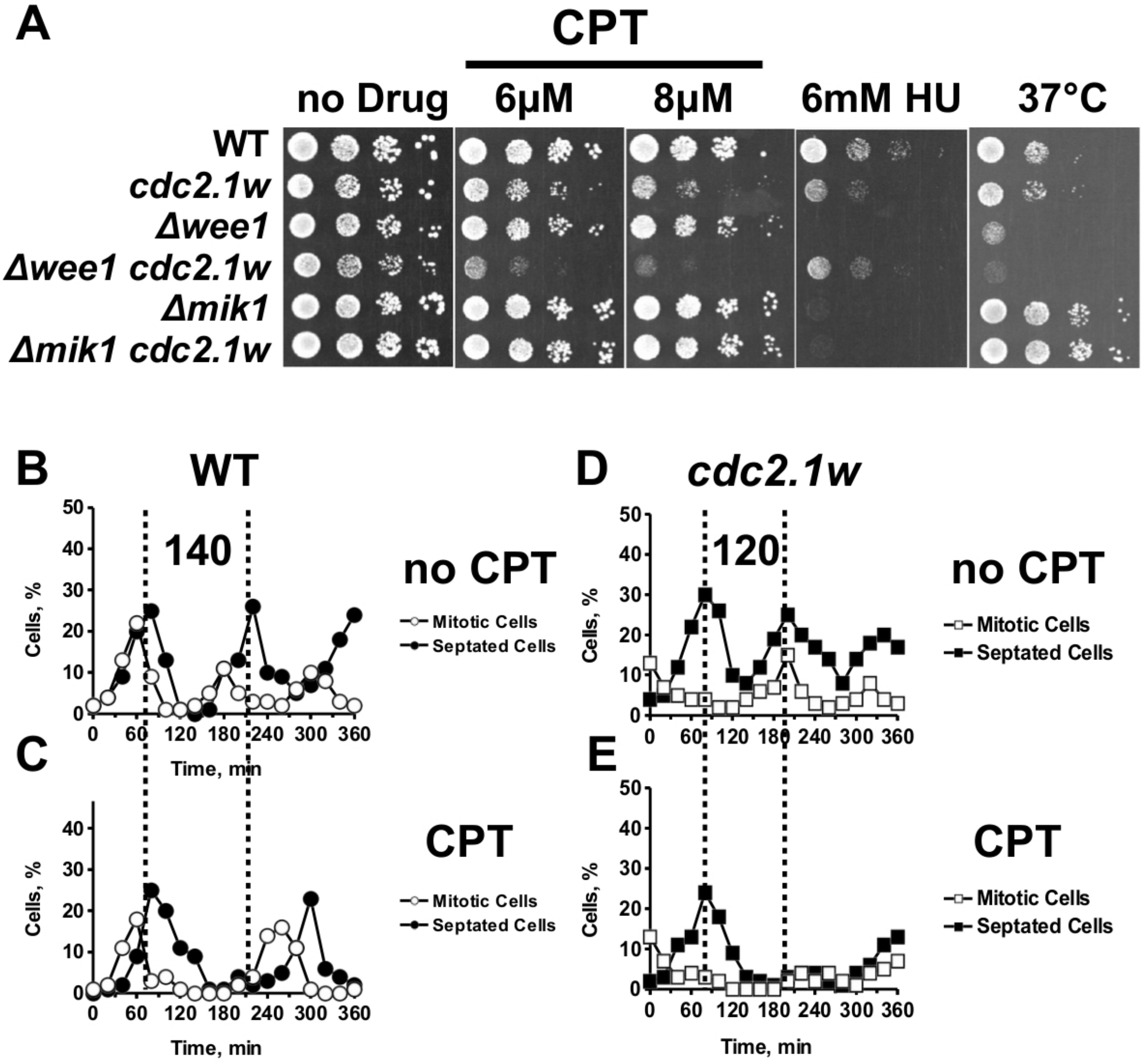

Supplementary Figure 1: (**A**) Wee1 and Cdc2.1w act in the same CPT response pathway**.** Drug sensitivity of wild type, *cdc2.1w*,*Δwee1*, *Δwee1 cdc2.1w*, *Δmik1* and *Δmik1 cdc2.1w* cells. Serial dilutions (10-fold; starting with 10^7 cells/ml) of the listed strains were spotted onto YEA plates containing the indicated drugs. The plates were incubated at 30°C for 3 days. One YEA plate was incubated at 37°C. **(B-E)** G2 phase is 20min shorter in *cdc2.1w* cells. The indicated strains were synchronised by lactose gradient centrifugation in early G2 and released into YEA medium with or without 40µM CPT at 30°C. 40µl aliquots were withdrawn in 20min intervals and added to 300µl methanol. Cells were stained with hoechst (1:1000) and calcofluor (1:100) (calcoflour 1mg/ml in 50mM sodium citrate, 100mM sodium phosphate pH 6.0; hoechst 10mg/ml in water) prior to scoring under a fluorescence microscope. Panels B and C show wild type cells in the absence (B) and presence of CPT (C). Panels D and E show *cdc2.1w* cells in the absence (D) and presence of CPT (E). Open symbols: bi-nucleated mitotic cells, closed symbols: septated G1/S cells.
